# Phylogenetic analyses of *Candidatus* Branchiomonas cysticola refine the taxonomic classification of Betaproteobacteria associated with epitheliocystis in fish

**DOI:** 10.1007/s00203-023-03581-1

**Published:** 2023-05-13

**Authors:** Even Bysveen Mjølnerød, Erwan Lagadec, Are Nylund

**Affiliations:** Fish Disease Research Group, Department of Biological Sciences, University of Bergen, PO Box 7803, 5020 Bergen, Norway

**Keywords:** *Candidatus* Branchiomonas cysticola, Epitheliocystis, Phylogeny, MLSA, Relative evolutionary divergence, 16S rRNA

## Abstract

*Candidatus* Branchiomonas cysticola is recognized as the most prevalent bacterial agent causing epitheliocystis in Atlantic salmon (*Salmo salar*). Based on its partial 16S rRNA sequence, the bacterium has previously been found to be a member of Burkholderiales in the class Betaproteobacteria. Multilocus Sequence Analysis (MLSA) of the bacterium and 60 type strains of Betaproteobacteria using newly identified housekeeping genes (*dnaK, rpoC,* and *fusA*) and ribosomal subunit sequences (16S and 23S), instead supported the bacterium’s affiliation to Nitrosomodales. Taxonomic rank normalization by Relative Evolutionary Divergence (RED) showed the phylogenetic distinction between *Cand.* B. cysticola and its closest related type strain to be at the family level. A novel bacterial family named *Branchiomonaceae* has thus been proposed to include a monophyletic clade of Betaproteobacteria exclusively associated with epitheliocystis in fish.

**Supplementary Information:**

The online version contains supplementary material available at 10.1007/s00203-023-03581-1.

## Introduction

Epitheliocystis found on gills of a wide range of fish species in both fresh- and sea water is caused by intracellular, intravacuolar bacteria, but none have been successfully cultured in vitro on agar (Nylund et al. 1998; Nowak and LaPatra 2006) Hence, all epitheliocystis-causing bacteria have *Candidatus* species status, and their identification are in most cases based on morphology and 16S rRNA sequences only. Several bacteria, all members of Chlamydiales (*Cand*. Piscichlamydia salmonis, *Cand*. Clavichlamydia salmonicola, and *Cand*. Syngnamydia salmonis) have been found associated with epitheliocystis in Atlantic salmon (*Salmo salar*) (Nylund et al. 1998, 2015; Draghi et al. 2004; Karlsen et al. 2008).

The epitheliocystis-causing bacterium *Candidatus* Branchiomonas cysticola present in salmon gills was identified as a member of Betaproteobacteria based on morphology, 16S rRNA sequence, and fluorescent in situ hybridization (Toenshoff et al. 2012). According to the authors, *Cand*. B. cysticola was the most abundant bacteria causing epitheliocystis on the gills of salmon, and ultrastructural studies of the cysts showed that elementary bodies, characteristic of the chlamydia life cycle, were lacking. Multiple studies have since found *Cand*. B. cysticola to be the dominant epitheliocystis-causing agent present on salmon gills, and when abundant, is associated with serious gill pathology (Steinum et al. 2010; Mitchell et al. 2013; Wiik-Nielsen et al. 2017; Gjessing et al. 2019, 2021). Recent studies also suggest that *Cand.* B. cysticola plays a central role in the development of complex gill disease (CGD) in Norwegian aquaculture (Herrero et al. 2018; Gjessing et al. 2019, 2021; Boerlage et al. 2020).

The relationship of *Cand*. B. cysticola with respect to other Betaproteobacteria is at present exclusively based on the partial 16S rRNA gene sequence (Toenshoff et al. 2012). However, recent identification of partial housekeeping (HK) gene sequences (Mjølnerød et al. 2022) and the complete rRNA operon of this bacterium, enable a more thorough analysis of the bacterium’s interspecific relationship within this class of bacteria. The closest relative, based on 16S rRNA, has been identified as an epitheliocystis-causing bacterium (clone BK-BJC) in lake trout (*Salvelinus namaycush*) (Contador et al. 2016). Other Betaproteobacteria, *Cand*. Ichthyocystis spp., causing epitheliocystis in fish species, have also been identified, and based on 16S rRNA analyses these bacteria represent close relatives to *Cand*. B. cysticola (Seth-Smith et al. 2016; Cascarano et al. 2022; Cascarano and Katharios 2022). Even though 16S rRNA is the only common sequence information available from these *Candidatus* species, it could be assumed that they all belong to the same family within Betaproteobacteria.

The continuous expansion of epitheliocystis outbreaks associated with Betaproteobacteria in fish species worldwide (Contador et al. 2016; Seth-Smith et al. 2016; Cascarano et al. 2022; Cascarano and Katharios 2022), stresses the need for a clarification/refining of the taxonomic affiliations of these bacteria. We hence propose a revised taxonomic classification of *Cand*. B. cysticola using MLSA and taxonomic rank normalization by RED (Parks et al. 2018).

## Materials and methods

### Gene mining, multilocus sequence analysis and 16S rRNA phylogeny

*Candidatus* B. cysticola-specific ribosomal RNA (16S and 23S) and partial HK gene sequences (elongation factor G, *fusA,* DNA-directed RNA-polymerase subunit β, *rpoC*, and heat shock protein-70, *dnaK*) were identified from a whole genome shotgun sequencing project (JAKNSC010000000), and verified as described by Mjølnerød et al. (2022) (accession numbers: OQ077712-OQ077714, OL314810.1, OP962438). Whole genome sequences of 60 type strains of Betaproteobacteria (Supplementary Table 1) were imported into Geneious Prime (2021) from the National Centre for Biotechnology Information (NCBI) genome database. *Pseudomonas aeruginosa* PAO1^T^, *Bacillus cerus* strain BC33^T^, *Clostridium perfringens* ATCC 13124^ T^, *Flavobacterium psychrophilum* 1604 1 5N^T^ and *Tenuifilum thalassicum* strain 38H^T^ were included as an outgroup for phylogenetic analyses.

Genes encoding 16S rRNA, 23S rRNA, elongation factor G, DNA-directed RNA-polymerase subunit β, and heat shock protein-70 were retrieved from each type strain’s genome and subsequently aligned in Geneious Prime using MAFFT v7.490 (Katoh et al. 2002; Katoh and Standley 2013). The sequence alignment algorithm chosen was L-INS-I using a default scoring matrix (200PAM/*k* = 2), gap open penalty (1.53) and offset value (0.123). Alignments were exported in a FASTA format before being re-imported in FASconCAT-G v1.05 (Kück and Longo 2014) to produce a relaxed PHYLIP super-matrix of the concatenated sequence alignments. Optimal partitioning scheme and substitution models for phylogenetic analysis was subsequently performed using PartitionFinder2 (Lanfear et al. 2017).

The concatenated super-matrix was reformatted to a NEXUS format before the data block containing parameters for best fit partition scheme and substitution models from PartitionFinder2 was inserted. Substitution model GTR + I + G was found to be the optimal nucleotide substitution model for all subsets of the alignment for Bayesian inferred phylogenetic analysis. Markov chain Monte Carlo (MCMC) parameters for number of; generations (ngens = 10,000,000), chains (nchains = 4), and temperature (temp = 0.15) were included to the NEXUS file that ultimately was subject to Bayesian analysis using MrBayes v.3.2.7a (Ronquist and Huelsenbeck 2003).

Convergence between the independent runs was assumed reached as the average standard deviation of split frequencies (SDSF) after 10,000,000 generations was < 0.01. Posterior analysis of the MCMC to confirm that the chains had reach stationary was performed using Tracer v1.7.2 (Rambaut et al. 2018). Graphical visualization and export of the consensus tree generated from MrBayes was done in FigTree v1.4.4 (Rambaut 2012) and Adobe Illustrator v26.5.2. Annotated fish icons were downloaded from free online resources of non-copyrighted SVG files.

A single locus 16S rRNA Bayesian phylogenetic analysis including 37 representative type strains of Betaproteobacteria and 17 bacterial strains associated with epitheliocystis in fish (Contador et al. 2016; Seth-Smith et al. 2016; Cascarano et al. 2022; Cascarano and Katharios 2022) was conducted in MrBayes. *Pseudomonas aeruginosa* strain PAO1^T^ was used as an outgroup. Data preparation, substitution model test and MCMC analysis was performed as for the MLSA, except the number of generations which was restricted to 5,000,000 generations.

### Pairwise distance matrix and relative evolutionary divergence

A distance matrix of the pairwise nucleotide identity (PNI) between type strains of Betaproteobacteria included in the analysis was exported from the concatenated alignment in Geneious Prime. The matrix was subsequently imported into RStudio 2021.09.1 (Team 2021) for graphical visualization. Dendrogram and heatmap representation of the pairwise distance matrix was performed using the “heatmap.2” function in package “ggplot2” version 3.3.5 (Wickham 2016). Incorporation of taxonomic information from NCBI and subsequent calculation of RED values for taxa subject to MLSA was performed using PhyloRank v.0.1.12 https://github.com/dparks1134/PhyloRank (Parks et al. 2018).

## Results

### Multi-locus sequence analysis

**Fig. 1:**
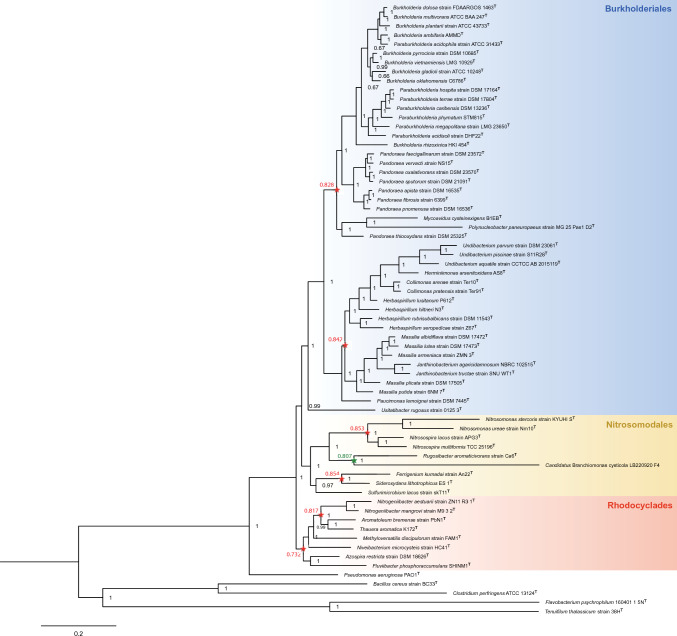
Bayesian inferred phylogenetic tree of 61 type strains of Betaproteobacteria and *Candidatus* Branchiomonas cysticola LB220920 F4 based on concatenated sequences of 16S rRNA, 23S rRNA, *dnaK*, *fusA* and *rpoC* gene sequences (8819 bp). *Pseudomonas aeruginosa* PAO1^T^, *Bacillus cerus* strain BC33, *Clostridium perfringens* ATCC 13124^ T^, *Flavobacterium psychrophilum* 1604 1 5N^T^ and *Tenuifilum thalassicum* strain 38H^T^ represent the outgroup. Branch support values are indicated at each node in black. Each familial node (star) and its respective RED are indicated in red color. The shared node and RED of *Cand*. B. cysticola and *Rugosibacter aromoaticivoran* strain Ca6^T^ is indicated in green. The order affiliations for each taxon of Betaproteobacteria are highlighted in colored boxes (colour figure online)

Phylogenetic reconstruction of 61 strains of Betaproteobacteria based on the concatenated (16S rRNA, 23S rRNA, *dnaK, fusA,* and *rpoC*) gene sequences (8819 bp) revealed *Cand.* B. cysticola as a phylogenetic distant taxon in this class of bacteria. Strong branch support values show the bacterium’s putative affiliation to the order of Nitrosomodales (Fig. 1). The type strain of closest evolutionary relationship to *Cand*. B. cysticola was found to be *Rugosibacter aromaticivorans* strain Ca6^T^, though still being considerably genetically disparate from each other.

### Pairwise distance matrix

**Fig. 2:**
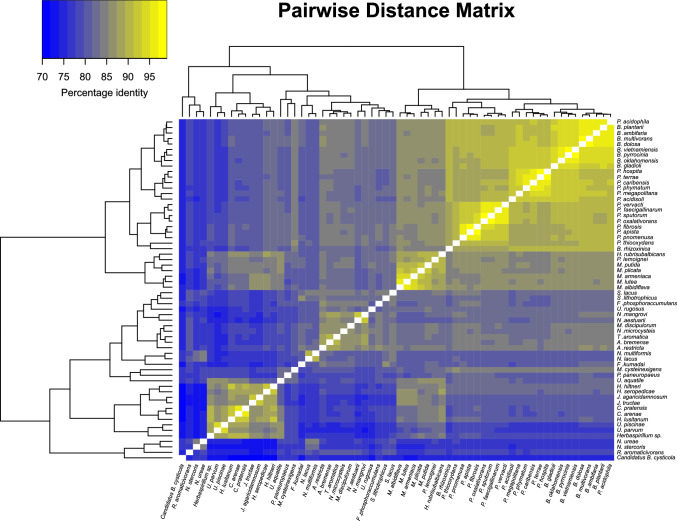
Pairwise distance matrix of the concatenated 16S rRNA, 23S rRNA, *dnaK*, *fusA*, and *rpoC* gene sequences of 60 type strains of *Betaproteobacteria* and *Cand.* B. cysticola. Dendrograms and heatmap show the PNI between all species

The pairwise distance matrix comprising all type strains of Betaproteobacteria subject to analysis demonstrated the unique genotype represented by *Cand.* B. cysticola. The average PNI of the concatenated sequences between this bacterium and the respective type strains of Betaproteobacteria was 74.03% (Fig. 2). Excluding *Cand.* B. cysticola, the average PNI of the total sample set was 83.01%). Despite displaying the closest evolutionary relationship, *Rugosibacter aromaticivorans* strain Ca6^T^ still only shared 75.36% nucleotide identity with *Cand.* B. cysticola.

### Taxonomic rank normalization

**Fig. 3:**
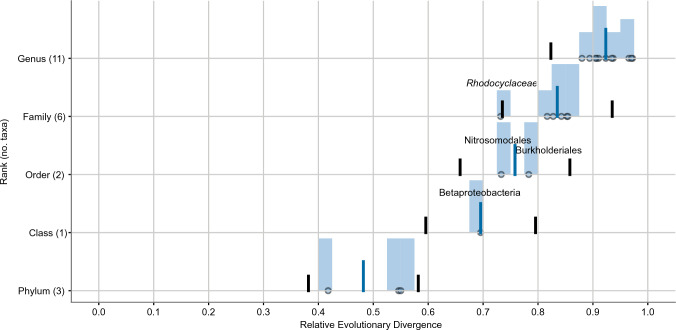
Relative Evolutionary Divergence (RED) plot of NCBI taxa from Bayesian inferred phylogenetic reconstruction based on the 16S rRNA, 23S rRNA, *dnaK*, *fusA*, and *rpoC* gene sequences. Median RED for each taxonomic rank is shown by the blue line and the rank intervals (± 0.1) are shown by the black lines. The blue histogram overlaying each point corresponds to the density of its corresponding monophyletic taxa. Names of notable taxa are annotated over their respective points in the plot

Relative evolutionary divergence values of the taxonomic ranks represented in the analysis all fell within the intervals for normalization (median RED for each rank ± 0.1, Fig. 3) except for *Rhodocyclaceae*. Median RED for the families present in the phylogenetic analysis of concatenated sequences was 0.835 (Fig. 3). *Rhodocyclaceae* displayed a RED of 0.732 (Figs. 1, 3) and is consequently just outside its rank interval. In comparison, the ancestral node of *Rugosibacter aromaticivorans* Ca6^T^ and *Cand.* B. cysticola had a RED of 0.807 (Fig. 1).

### 16S rRNA phylogeny

**Fig. 4:**
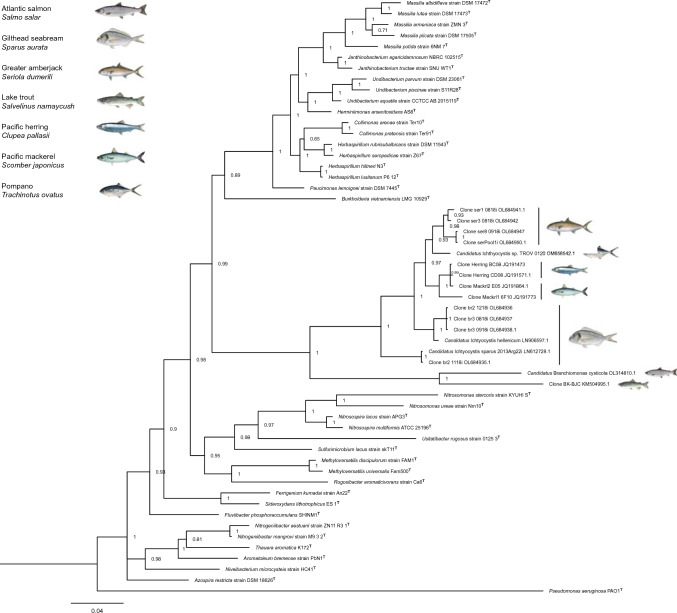
Bayesian inferred phylogenetic tree based on the 16S rRNA sequence of 37 representative type strains of Betaproteobacteria and 17 bacterial strains associated with epitheliocystis. Fish symbols indicate which fish species each sequence has been retrieved from. The gammaproteobacterium *Pseudomonas aeruginosa* strain PAO1^T^ represents the outgroup. Branch support values are indicated at each node

Phylogenetic analysis of the 16S rRNA sequences resulted in a monophyletic clade of bacteria exclusively associated with epitheliocystis in fish (Fig. 4). Two separate lineages were identified within the clade, where sequences closely related to the *Ichthyocystis* genus made up the more comprehensive of the two. Clone BK-BJC from lake trout and *Cand*. B. cysticola from Atlantic salmon comprised the second, slightly more divergent lineage.

## Discussion

The taxonomic classification of *Cand.* B. cysticola associated with epitheliocystis in farmed Atlantic salmon, has since its first description been exclusively based on its 16S rRNA gene sequence (Toenshoff et al. 2012). By applying three different methods of phylogenetic analysis (maximum parsimony, maximum likelihood, and Neighbor-Joining), the bacterium was found to constitute a deep branching lineage within Betaproteobacteria*.* However, as described by the authors, low bootstrap values and inconsistent branching dependent on the methods applied, did not allow reliable elucidation of its phylogenetic relationship within this class. Despite the limited discriminatory power of 16S rRNA, this analysis has until now served as the basis for the taxonomic classification of *Cand.* B. cysticola. MLSA of partial sequences of newly identified HK genes and of the rRNA operon of this bacterium, has now enabled a phylogenetic reconstruction with increased resolution in the description of the bacterium’s affiliation to Betaproteobacteria*.*

Betaproteobacteria associated with epitheliocystis in fish have previously been described from lake trout (*Salvelinus namaycush*) in Ontario, Canada (Contador et al. 2016) and gilthead seabream (*Sparus aurata*) from Greece (Seth-Smith et al. 2016). Two species were ultimately characterized in the novel *Candidatus* genus of *Ichtyocystis* by Seth-Smith et al. (2016) (*Cand.* Ichtyocystis sparus and *Cand.* Ichtyocystis hellenicum). Recent studies have identified new agents of this genus associated with epitheliocystis in reared greater amberjack (*Seriola dumerili*), wild-caught Pacific herring (*Clupea pallasii*) and Pacific mackerel (*Scomber japonicus*) (Cascarano et al. 2022), as well as from pompano (*Trachinotus ovatus*) (Cascarano and Katharios 2022).

Like *Cand.* B. cysticola, species of the *Ichtyocystis* genus have been classified as members of the order Burkholderiales (Toenshoff et al. 2012; Mitchell et al. 2013; Seth-Smith et al. 2016). Phylogenetic reconstruction based on the concatenation of 16S, 23S, *dnaK*, *fusA*, and *rpoC* gene sequences, however, strongly supports *Cand.* B. cysticola’s affiliation to Nitrosomodales (Fig. 1)*.* The bacterium’s considerable phylogenetic distinction (Fig. 1) and its unique nucleotide identity compared to other type strains of its class (Fig. 2), prompted an effort to resolve the taxonomic ranks of the bacterium through RED.

Taxonomic rank normalization by RED was consistent with the NCBI taxonomy, except for *Rhodocyclaceae* (Figs. 1, 3) that was marginally outside its rank interval. Noticably, the order Rhodocyclales was not plotted in the rank normalization plot (Fig. 3) as Phylorank only plots taxa with two or more subordinate taxa (Parks et al. 2018). However, as Rhodocyclales and *Rhodocyclaceae* share the same internal node, the RED value of the former is also thought to be 0.732 (Fig. 1). This is similar to the values of Burkholderiales and Nitrosomodales (0.783 and 0.733, respectively), and well within its rank intervals (Fig. 3). The internal node of *Cand.* B. cysticola and *Rugosibacter aromaticivorans* strain Ca6^T^, on the other hand, was found to display a lower RED value (0.807, Fig. 1) than the median of families represented in the analysis (Fig. 3). The evolutionary divergence between the two species is accordingly at the family level and thus support the description of separate families within Nitrosomodales.

*Rugosibacter aromaticivorans* is an extracellular bacterium isolated from contaminated soil and found to be a member of *Rhodocyclaceae* in the order Rhodocyclales, based on its 16S rRNA sequence (Corteselli et al. 2017). Discrepancies from its original classification were therefore found as the MLSA suggests it constitutes a member of Nitrosomodales (Fig. 1). Phylogenetic analysis of the 16S rRNA sequence did, nonetheless, show this bacterium’s relationship to members of Rhodocyclales (Fig. 4) to be in accordance with its previous description. However, reassigning the taxonomic affiliation of *R. aromaticivorans* is not the objective of the present study. However, the rapid expansion of Betaproteobacteria associated with epitheliocystis in farmed and wild fish species worldwide, calls for the establishment of taxonomic ranks designated to these unique bacterial species.

The 16S rRNA phylogeny including betaproteobacterial strains found to cause epitheliocystis in fish, formed a monophyletic clade of these closely associated bacteria (Fig. 4). This is in compliance with previous phylogenies using 16S rRNA sequences (Contador et al. 2016; Seth-Smith et al. 2016; Cascarano and Katharios 2022; Cascarano et al. 2022). Consequently, it is reasonable to believe that all taxa of this clade share the same common ancestor of *Cand*. B. cysticola and *R. aromaticivorans* described by MLSA. We, therefore, propose that the ancestral node (RED = 0.807) and the descendant branch of *Cand.* B. cysticola (Fig. 1) represents the divergence of a novel bacterial family to be named *Branchiomonaceae*. The name is derived from the type genus of this novel family; *Branchiomonas*, originally named by Toenshoff et al., (2012) which combines the gr. noun branchia (gills) and gr. noun monas (a unit, monad). The proposal of this novel bacterial family will hopefully aid future taxonomic classifications of Betaproteobacteria associated with epiteliocystis in fish species worldwide.

## Supplementary Information

Below is the link to the electronic supplementary material.

Supplementary file1 (XLSX 12 KB)

## Data Availability

The sequence data generated during the current study are available in the Genbank repository under accession numbers (OQ077712-OQ077714, OL314810.1, and OP962438). Genbank accession numbers for the whole genome sequences of the separate type strains used in the present study are listed in Supplementary Table 1.
